# Maternal Respiratory Syncytial Virus Vaccination and Receipt of Respiratory Syncytial Virus Antibody (Nirsevimab) by Infants Aged <8 Months — United States, April 2024

**DOI:** 10.15585/mmwr.mm7338a2

**Published:** 2024-09-26

**Authors:** Hilda Razzaghi, Emma Garacci, Katherine E. Kahn, Megan C. Lindley, Jefferson M. Jones, Shannon Stokley, Kayla Calhoun, Carla L. Black

**Affiliations:** ^1^Immunization Services Division, National Center for Immunization and Respiratory Diseases, CDC; ^2^Cherokee Nation Operational Solutions, Tulsa, Oklahoma; ^3^Eagle Health Analytics, Atlanta, Georgia; ^4^Coronavirus and Other Respiratory Viruses Division, National Center for Immunization and Respiratory Diseases, CDC.

**Summary**

**What is already known about this topic?**

Respiratory syncytial virus (RSV) is the most common cause of hospitalization among U.S. infants. Receipt of either a maternal RSV vaccine or administration of RSV antibody (nirsevimab) to infants was first recommended during the 2023–24 RSV season.

**What is added by this report?**

In a CDC survey, 33% of eligible pregnant women reported receiving an RSV vaccination. Among women with a live birth, 45% reported that their infant received nirsevimab. Overall, 56% of infants were protected against severe RSV disease by either product or both. Provider recommendation for immunization was associated with higher coverage.

**What are the implications for public health practice?**

Enhanced measures to implement provider RSV immunization recommendations are needed to protect infants from severe RSV disease.

## Abstract

Respiratory syncytial virus (RSV) is the most common cause of hospitalization among U.S. infants. CDC recommends RSV vaccination for pregnant persons or administration of RSV antibody (nirsevimab) to infants aged <8 months to prevent RSV lower respiratory tract disease among infants. To determine maternal and infant RSV immunization coverage for the 2023–24 RSV season, CDC conducted an Internet panel survey during March 26–April 11, 2024. Among 678 women at 32–36 weeks’ gestation during September 2023–January 2024, 32.6% reported receipt of an RSV vaccine any time during pregnancy. Among 866 women with an infant born during August 2023–March 2024, 44.6% reported receipt of nirsevimab by the infant. Overall, 55.8% of infants were protected by maternal RSV vaccine, nirsevimab, or both. Provider recommendation for maternal vaccination or infant nirsevimab was associated with higher immunization coverage, whereas lack of a provider recommendation was the main reason for not getting RSV immunization. The main reason for definitely or probably not getting nirsevimab for infants was concern about the long-term safety for the infant. Activities supporting providers to make RSV prevention recommendations and have informative conversations with patients might increase the proportion of infants protected against severe RSV disease. CDC and the American College of Obstetricians and Gynecologists have resources to assist providers in effectively communicating the importance of immunization.

## Introduction

Respiratory syncytial virus (RSV) is the most common cause of hospitalization among U.S. infants, with the highest RSV-associated hospitalization incidence among infants aged <3 months (*1*). To protect all infants against RSV-associated lower respiratory tract disease, the Advisory Committee on Immunization Practices (ACIP) recommends a single lifetime dose of RSV vaccine for pregnant persons at 32–36 weeks’ gestation using seasonal administration during September–January (*2*) or administration of nirsevimab, an RSV antibody, for infants aged <8 months born during or entering their first RSV season during October–March in most of the continental United States (*3*). For most infants, both products are not needed except in rare circumstances (*2*). The 2023–24 RSV season was the first season during which maternal RSV vaccination and nirsevimab for infants were recommended for prevention of severe infant RSV disease. This report provides estimates of RSV maternal vaccination coverage, receipt of nirsevimab by infants, and proportion of infants protected by either product in the United States during the 2023–24 RSV season (*4*).

## Methods

### Data Source and Study Participants

CDC conducted an Internet panel* survey during March 26–April 11, 2024, to determine end-of-season influenza vaccination coverage among pregnant women, as previously described (*5*). Questions about maternal RSV vaccination and nirsevimab administration for infants were included. Women aged 18–49 years who reported being pregnant at any time since August 1, 2023, were eligible for the survey. Among 2,473 eligible women, 2,266 (91.6%)^†^ completed the survey. The final analytic sample included 2,263 currently and recently pregnant women. Data were weighted to reflect pregnancy status and outcome at the time of survey completion, age, race and ethnicity, and geographic distribution of the total U.S. population of pregnant women.

### Data Analysis

Analysis of RSV vaccination coverage among pregnant and recently pregnant women was limited to 678 women who were 32–36 gestational weeks’ pregnant any time during September 1, 2023–January 31, 2024. Women who reported receipt of RSV vaccine during pregnancy were considered vaccinated regardless of timing of vaccination. Analysis of nirsevimab coverage among infants as well as proportion of infants protected by either maternal or infant immunization was evaluated among 866^§^ women who had a live birth during August 1, 2023–March 31, 2024, whose infants would have been eligible to receive nirsevimab during October 1, 2023–March 31, 2024. Infants were considered protected against severe RSV disease if mothers reported either receipt of maternal RSV vaccination or receipt of nirsevimab by the infant. The analysis of immunization preference for maternal RSV vaccine or infant nirsevimab included 2,023 currently pregnant and recently pregnant women who had a live birth.

SAS (version 9.4, SAS institute) and SAS-callable SUDAAN software (version 11.0.4; RTI International) were used to conduct all analyses. Weighted proportions and corresponding 95% CIs for maternal and infant RSV immunization coverage were estimated overall and by selected demographic characteristics. Differences among groups were determined using *t*-tests, with p-values <0.05 considered statistically significant. This activity was reviewed by CDC, deemed research not involving human subjects, and was conducted consistent with applicable federal law and CDC policy.^¶^

## Results

### Maternal RSV Vaccination Coverage

Among 678 eligible women, maternal RSV vaccination coverage was 32.6% overall and was significantly higher among those with private or military insurance (38.9%) than among those with public insurance (28.0%); those living at or above poverty (35.0%) compared with those living below poverty (26.4%); those with higher than a college degree (50.1%) than among those with a college degree or less (28.7%–32.7%); and among those who received a provider recommendation for either maternal or infant RSV immunization (56.7%) than among those who received no recommendation (1.9%) (Table 1). The majority of vaccinated women (54.1%) reported receiving the vaccine at an obstetrician or gynecologist’s office.

**TABLE 1:** Respiratory syncytial virus vaccination and respiratory syncytial virus antibody (nirsevimab) coverage among pregnant women and their infants, by selected characteristics — Internet panel survey, United States, April 2024

Characteristic	Maternal RSV vaccination*		Receipt of nirsevimab by infant^†^		Maternal RSV vaccination or receipt of nirsevimab by infant^§^	
	Total no. (weighted %)^¶^	Weighted % vaccinated (95% CI)**	Total no. (weighted %)^¶^	Weighted % vaccinated (95% CI)**	Total no. (weighted %)^¶^	Weighted % vaccinated (95% CI)**
**Overall**	**678**	**32.6 (28.8–36.6)**	**866**	**44.6 (40.9–48.3)**	**866**	**55.8 (52.1–59.6)**
**Maternal age group, yrs**						
35–49 (Ref)	**181 (17.6)**	37.4 (29.8–45.5)	**257 (20.6)**	48.4 (41.8–55.2)	**257 (20.6)**	61.3 (54.5–67.7)
25–34	**378 (60.8)**	28.9 (24.1–34.1)	**466 (58.9)**	42.5 (37.6–47.6)	**466 (58.9)**	54.0 (48.9–59.0)
18–24	**119 (21.5)**	39.1 (29.7–49.2)	**143 (20.5)**	46.5 (37.5–55.7)	**143 (20.5)**	55.8 (46.6–64.8)
**Race and ethnicity^††^**						
Black or African American	**110 (14.5)**	36.4 (26.6–47.0)	**129 (14.0)**	51.1 (41.4–60.7)	**129 (14.0)**	56.5 (46.8–65.9)
White (Ref)	**396 (49.5)**	33.8 (28.8–39.0)	**524 (51.2)**	44.2 (39.7–48.8)	**524 (51.2)**	57.8 (53.2–62.3)
Hispanic or Latino	**121 (26.5)**	29.3 (21.2–38.6)	**147 (25.7)**	43.9 (35.1–53.0)	**147 (25.7)**	53.7 (44.7–62.6)
Other	**51 (9.4)**	29.9 (16.5–46.3)	**66 (9.1)**	38.5 (25.9–52.3)	**66 (9.1)**	50.0 (35.7–64.2)
**Maternal education**						
Higher than college degree (Ref)	**86 (10.6)**	50.1 (38.1–62.0)	**112 (11.3)**	37.2 (27.8–47.5)	**112 (11.3)**	63.4 (53.0–72.8)
College degree	**234 (33.6)**	32.7 (26.2–39.7)^§§^	**302 (33.8)**	46.2 (39.9–52.5)	**302 (33.8)**	55.9 (49.5–62.1)
Some college, no degree	**169 (23.2)**	30.0 (22.9–38.0)^§§^	**220 (24.6)**	45.3 (38.0–52.7)	**220 (24.6)**	55.2 (47.7–62.6)
High school diploma or less	**189 (32.6)**	28.7 (21.8–36.4)^§§^	**232 (30.4)**	45.0 (37.7–52.4)	**232 (30.4)**	53.5 (46.0–60.9)
**Maternal employment status**						
Employed (Ref)	**410 (59.1)**	35.7 (30.7–40.9)	**531 (59.8)**	48.5 (43.8–53.2)	**531 (59.8)**	60.1 (55.3–64.7)
Unemployed	**268 (40.9)**	28.2 (22.2–34.7)	**335 (40.2)**	38.7 (32.9–44.8)^§§^	**335 (40.2)**	49.6 (43.5–55.7)^§§^
**Poverty status^¶¶^**						
At or above poverty (Ref)	**496 (72.1)**	35.0 (30.4–39.8)	**647 (73.3)**	44.5 (40.2–48.8)	**647 (73.3)**	57.3 (53.0–61.6)
Below poverty	**182 (27.9)**	26.4 (19.8–33.9)^§§^	**219 (26.7)**	44.7 (37.4–52.2)	**219 (26.7)**	51.7 (44.2–59.3)
Area of residence***						
Nonrural (Ref)	**536 (79.7)**	33.1 (28.8–37.7)	**693 (81.2)**	44.7 (40.6–48.9)	**693 (81.2)**	56.2 (52.0–60.4)
Rural	**142 (20.3)**	30.5 (22.4–39.7)	**173 (18.8)**	43.9 (35.8–52.3)	**173 (18.8)**	54.3 (45.7–62.7)
**Region^†††^**						
Northeast (Ref)	**86 (15.1)**	38.6 (27.4–50.8)	**116 (15.9)**	44.0 (34.2–54.2)	**116 (15.9)**	54.3 (44.1–64.3)
Midwest	**163 (19.5)**	36.8 (29.0–45.2)	**212 (20.2)**	48.7 (41.2–56.1)	**212 (20.2)**	61.2 (53.6–68.3)
South	**299 (40.3)**	29.9 (24.3–35.9)	**379 (40.5)**	47.4 (41.8–53.0)	**379 (40.5)**	56.5 (50.9–62.0)
West	**130 (25.0)**	30.1 (21.9–39.3)	**159 (23.5)**	36.6 (28.5–45.2)	**159 (23.5)**	51.2 (42.3–60.1)
**Prenatal insurance coverage^§§§^**						
Private or military insurance only (Ref)	**313 (41.7)**	38.9 (32.9–45.1)	**417 (45.1)**	43.1 (37.9–48.4)	**417 (45.1)**	58.9 (53.6–64.1)
Any public insurance	**339 (53.8)**	28.0 (22.9–33.6)^§§^	**418 (50.9)**	46.9 (41.6–52.3)	**418 (50.9)**	53.7 (48.3–59.1)
No insurance	**26 (4.4)**	—^¶¶¶^	**31 (4.0)**	—^¶¶¶^	**31 (4.0)**	—^¶¶¶^
Provider recommendation of RSV vaccination or nirsevimab administration****						
Recommendation (Ref)	**388 (56.0)**	56.7 (51.1–62.2)	**469 (53.5)**	58.7 (53.6–63.7)	**469 (53.5)**	79.2 (74.6–83.3)
No recommendation	**290 (44.0)**	1.9 (0.6–4.4)^§§^	**397 (46.5)**	28.3 (23.5–33.5)^§§^	**397 (46.5)**	29.0 (24.2–34.2)^§§^
**Timing of maternal RSV vaccination^††††^**						
Before 32 weeks’ gestation	**69 (29.7)**	NA	**NA**	NA	**NA**	NA
32–36 weeks’ gestation	**134 (58.4)**	NA	**NA**	NA	**NA**	NA
After 36 weeks’ gestation	**28 (11.9)**	NA	**NA**	NA	**NA**	NA
**Place of maternal RSV vaccination**						
Obstetrician, gynecologist, or midwife’s office	**124 (54.1)**	NA	**NA**	NA	**NA**	NA
Family physician or other physician's office	**23 (9.7)**	NA	**NA**	NA	**NA**	NA
Health department clinic	**14 (4.9)**	NA	**NA**	NA	**NA**	NA
Hospital	**28 (13.1)**	NA	**NA**	NA	**NA**	NA
Store (supermarket, drug store, or pharmacy)	**41 (18.0)**	NA	**NA**	NA	**NA**	NA
At work	**—^§§§§^ (0.2)**	NA	**NA**	NA	**NA**	NA

**Abbreviations:** NA = not applicable; Ref = referent group; RSV = respiratory syncytial virus.

* Respondents 32–36 gestational weeks pregnant any time during September 1, 2023–January 31, 2024, were included in the analyses. Women who reported receipt of an RSV vaccination any time during their pregnancy were considered immunized.

^†^ Infants born to survey participants during August 2023–March 2024 were included in the analysis. Infants with reported nirsevimab receipt were considered immunized. Although nirsevimab is indicated for infants aged <8 months during October 1–March 31, and thus born during February 2023–March 2024, the survey sample was based on influenza vaccination recommendations and only includes women whose pregnancy began August 1, 2023, or later.

^§^ Infants born to survey participants during August 2023–March 2024 were included in the analysis. Infants with reported nirsevimab receipt, or those born to women 32–36 weeks pregnant at any time during September 2023–January 2024 who reported receiving an RSV vaccination any time during their pregnancy, were considered immunized.

^¶^ The total unweighted number and weighted proportion of respondents in the sample.

** Modified Clopper-Pearson 95% CI according to the approach of Korn and Graubard. https://www150.statcan.gc.ca/n1/en/pub/12-001-x/1998002/article/4356-eng.pdf?st = nQVSWv1i

^††^ Race and ethnicity were self-reported. Respondents identifying as Hispanic or Latino might be of any race. The “Other” race category included Asian, American Indian or Alaska Native, Native Hawaiian or Pacific Islander, and women who selected multiple races.

^§§^ Statistically significant difference compared with Ref.

^¶¶^ Poverty status was defined based on the reported number of persons living in the household and annual household income, according to U.S. Census Bureau poverty thresholds. https://www.census.gov/data/tables/time-series/demo/income-poverty/historical-poverty-thresholds.html

*** Rurality was defined using zip code areas in which >50% of the population lives in a nonmetropolitan county, a rural U.S. Census Bureau tract, or both, according to the Health Resources and Services Administration’s definition of rural population. https://www.hrsa.gov/rural-health/about-us/definition/index.html

^†††^ https://www2.census.gov/geo/pdfs/maps-data/maps/reference/us_regdiv.pdf

^§§§^ Respondents pregnant on their survey date were asked what medical insurance or medical care coverage they had; respondents who had already delivered were asked what coverage they had during their most recent pregnancy. Women considered to have public insurance selected at least one of the following options: Medicaid, Medicare, state-sponsored medical plan, or other government plan. Respondents considered to have private or military insurance selected private medical insurance, military medical care, or both and did not select any type of public insurance.

^¶¶¶^ Estimates do not meet the National Center for Health Statistics’ standards of reliability. https://www.cdc.gov/nchs/data/series/sr_02/sr02_175.pdf

**** Respondents were asked, “During your current/most recent pregnancy, did any doctor, nurse, or other medical professional recommend that you get an RSV vaccination or that your baby receive an RSV antibody shot?”

^††††^ Among the 678 eligible pregnant women, 446 did not report receiving an RSV vaccine; one reported receipt of an RSV vaccine but not when the vaccine was received.

^§§§§^ Suppressed to avoid risk of disclosure.

### Infant Nirsevimab Coverage

Among 866 women with a live birth, infant coverage with nirsevimab was 44.6% overall and was significantly higher among infants whose mothers were employed (48.5%) than among those whose mothers were unemployed (38.7%) and among those who received a provider recommendation for either maternal or infant RSV immunization (58.7%) than among those who did not receive a recommendation (28.3%). Overall, 55.8% of infants were protected by either maternal RSV vaccination, nirsevimab, or both; 14.2% of infants were protected by both.

### Reasons for Nonvaccination

The most frequently reported main reasons for nonreceipt of maternal RSV vaccination were 1) not receiving a recommendation for vaccination from a doctor, nurse, or other medical professional (16.9%); 2) not knowing that RSV vaccination was needed during pregnancy (15.0%); and 3) having concerns about possible safety risks to the infant (12.0%) (Figure). Among unvaccinated pregnant and recently pregnant women with a live birth whose infant did not receive nirsevimab, the main reasons for definitely or probably not getting nirsevimab for their infants included 1) concerns about the long-term safety of nirsevimab for the infant (19.1%); 2) not planning to get the infant any vaccines (16.9%); and 3) not wanting the infant to receive too many vaccines (16.8%) (Figure).

**FIGURE:**
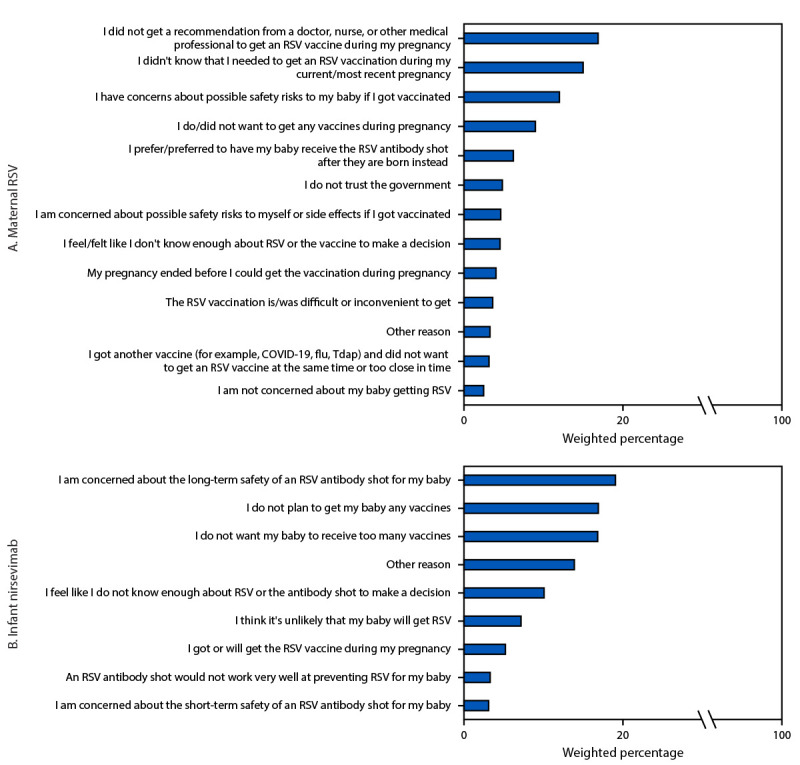
Main reason for not receiving respiratory syncytial virus vaccine among unvaccinated pregnant or recently pregnant women (N = 433) (A)* and probably or definitely not receiving respiratory syncytial virus antibody (nirsevimab) for unprotected infants (N = 240) (B)^†^^,^^§^ — Internet panel survey, United States, April 2024 **Abbreviations:** RSV = respiratory syncytial virus; Tdap = tetanus toxoid, reduced diphtheria toxoid, and acellular pertussis vaccine. * Currently or recently pregnant women with a live birth who were 32–36 gestational weeks pregnant any time during September 1, 2023–January 31, 2024, and were not vaccinated against RSV during their pregnancy, were asked to select from a list of reasons for not receiving a maternal RSV vaccine (among the 446 unvaccinated women, 11 recently pregnant women who did not report a live birth were not asked the question, and two did not respond to the question, leaving a sample size of 433). Respondents who selected more than one reason were asked to select the main reason. Reasons reported by <2% of respondents are not shown. ^†^ Currently pregnant women who were not vaccinated against RSV, and recently pregnant women with a live birth, who reported their infants had not received nirsevimab and would “definitely” or “probably” not receive nirsevimab, were asked to select from a list of reasons why their infant would “definitely” or “probably” not receive nirsevimab. Respondents who selected more than one reason were asked to select the main reason. Reasons reported by <2% of respondents are not shown. ^§^ Estimates for the responses “An RSV antibody shot would not work very well at preventing RSV for my baby” and “I am concerned about the short-term safety of an RSV antibody shot for my baby” do not meet the National Center for Health Statistics’ standards of reliability and should be interpreted with caution. https://www.cdc.gov/nchs/data/series/sr_02/sr02_175.pdf

### Maternal Product Preference

In determining product preference, 38.1% of respondents preferred to receive an RSV vaccine during pregnancy, 27.8% preferred nirsevimab for their infant, 21.3% had no preference, and 12.8% would not get the maternal RSV vaccine or nirsevimab for their infant (Table 2). Among those who preferred the maternal RSV vaccine, 47.8% believed that maternal vaccination would be safer, 30.2% were worried about their infant getting too many shots, and 30% believed that maternal vaccination would be more effective. Among those who preferred nirsevimab for their infant, 43.6% believed that it would be more effective, and 32.4% believed that it would be safer.

**TABLE 2:** Maternal preference for immunization by maternal respiratory syncytial virus vaccination or infant respiratory syncytial virus antibody (nirsevimab) among pregnant and recently pregnant women with a live birth,* and reasons for reported preference^†^ (N = 2,023) — Internet panel survey, United States, April 2024

Preference/Reason	No.	Weighted % (95% CI)^§^
**Maternal RSV vaccination during pregnancy**	782	38.1 (35.7–40.5)
I believe it will be safer	373	47.8 (43.8–51.9)
I believe it will be more effective	234	30.0 (26.4–33.8)
I am worried the antibody shot will not be available for my baby	105	12.4 (9.9–15.2)
I am worried the antibody shot will cost too much or not be covered by insurance	37	4.9 (3.3–6.9)
I am worried about my baby getting too many shots	225	30.2 (26.6–34.0)
I do not have enough information about the RSV antibody shot	127	16.1 (13.3–19.2)
Other reason	51	5.8 (4.2–7.8)
**RSV antibody shot for infant^§^**	576	27.8 (25.7–30.1)
I believe it will be safer	188	32.4 (28.0–36.9)
I believe it will be more effective	258	43.6 (39.0–48.3)
I am worried the vaccination will not be available during my pregnancy	46	7.5 (5.3–10.2)
I am worried the vaccination will cost too much or not be covered by insurance	41	7.3 (5.0–10.2)
I am worried about getting too many shots during my pregnancy	126	23.0 (19.1–27.3)
I do not have enough information about the RSV vaccination during pregnancy	108	18.8 (15.4–22.6)
Other reason	44	7.7 (5.5–10.5)
No preference	419	21.3 (19.3–23.4)
**I would not get an RSV vaccination for myself or the RSV antibody shot for my baby**	246	12.8 (11.1–14.5)

**Abbreviation:** RSV = respiratory syncytial virus.

* Currently and recently pregnant women with a live birth were asked the questions on preference.

^†^ Respondents could select more than one reason.

^§^ Modified Clopper-Pearson 95% CI according to the approach of Korn and Graubard. https://www150.statcan.gc.ca/n1/en/pub/12-001-x/1998002/article/4356-eng.pdf?st=nQVSWv1i.

## Discussion

This survey found that during the first season in which new immunization products were recommended to prevent RSV disease among infants, maternal RSV vaccination coverage among eligible pregnant women was 32.6%, and nirsevimab coverage among infants was 44.6%; 55.8% of infants were protected by either or both products. Receipt of a provider recommendation was strongly associated with both maternal and infant immunization.

Multiple challenges in rolling out the new immunization products might have resulted in lower than anticipated coverage. These challenges included timing of recommendations and product availability, differing recommendations in terms of timing of vaccine and nirsevimab administration, complex clinical considerations and nuanced communications, limited time to improve awareness of the new recommendations for both health care providers and pregnant women, cost and reimbursement issues, access issues, and concerns about safety and efficacy of the products (*6*). In addition, nirsevimab availability was limited during the 2023–24 RSV season, creating challenges in accessing maternal RSV vaccine that included cost and reimbursement and lack of supply at many obstetrics and gynecology offices (*6*,*7*). RSV immunization is most likely to prevent and decrease the risk for severe RSV disease when administered shortly before or at the beginning of the RSV season, but the limited availability of RSV products, particularly before the start and during the early months of the RSV season likely limited the effect of immunization during the 2023–24 season (*8*). Complexities in the RSV prevention recommendations, including the timing of vaccination of pregnant women and the recommendation for infant nirsevimab based on maternal RSV vaccination status, as well as the need for coordination of maternal and infant preventive care might also have contributed to an estimated 14.2% of infants reported to have received both nirsevimab and maternal RSV antibody protection, which is not indicated for most infants.

As observed for other immunizations, both receipt of maternal RSV vaccine and nirsevimab for infants was higher among those who received a provider recommendation for either product (*5*). Approximately one half of pregnant women did not report receiving a provider recommendation for maternal RSV vaccination or nirsevimab for their infants, indicating missed opportunities to protect infants from severe RSV disease. These findings further underscore the importance of a strong provider recommendation for immunization.

### Limitations

The findings in this report are subject to at least five limitations. First, this study consisted of a nonprobability sample, and results might not be generalizable to all pregnant women in the United States. Some self-selection bias or bias due to exclusion of women with no Internet access might have occurred. Second, maternal and infant immunization status was self-reported and might be subject to recall or social desirability bias. Maternal vaccination coverage estimates based on data from this survey were higher than were those based on electronic health record data from eight sites participating in the Vaccine Safety Datalink (VSD).** However, because VSD did not require continuous enrollment in the participating health care organizations for the duration of the pregnancy, recording of the vaccination status in VSD might have been incomplete, and the population served by the VSD sites might not be representative of the U.S. population of pregnant women (*9*,*10*). Nirsevimab coverage among infants in this study was similar to estimated coverage from the National Immunization Survey.^††^ Third, because of small sample sizes, immunization coverage could not be evaluated separately among some racial and ethnic groups. Fourth, statistical tests based on the assumption of probability were used to ascertain differences in immunization coverage among groups in this nonprobability sample. Finally, the survey sample is limited to infants born during August 2023–March 2024, and thus does not include all infants who would have been eligible for nirsevimab (i.e., those born during February 2023–March 2024).

### Implications for Public Health Practice

Recommendations from health care providers are critical to improving RSV immunization coverage for both pregnant women and their infants and reducing severe RSV disease among infants. CDC has resources to assist providers in effectively communicating the importance of vaccination, such as sharing specific reasons that recommended vaccines are right for the patient and highlighting positive personal or clinical experiences with vaccines.^§§^ In addition, the American College of Obstetricians and Gynecologists has an immunization toolkit^¶¶^ that includes communication strategies for providers. Expanded measures to implement RSV immunization recommendations are needed to protect infants from severe RSV disease.

## Footnotes

* Pregnant women were recruited from a large, preexisting, opt-in Internet panel of the general population, operated by Dynata. https://www.dynata.com/wp-content/uploads/2024/01/Dynata_Panel_Book.pdf

† A survey response rate requires specification of the denominator at each stage of sampling. During recruitment of an online opt-in survey sample, such as the Internet panels described in this report, these numbers are not available; therefore, a response rate cannot be calculated. Instead, the survey completion rate is provided.

§ Among the 866 women, 20 reported multiple births, but these were considered a single infant in the analysis.

¶ 45 C.F.R. part 46; 21 C.F.R. part 56; 42 U.S.C. Sect. 241(d); 5 U.S.C. Sect. 552a; 44 U.S.C. Sect. 3501 et seq.

**
https://www.cdc.gov/rsvvaxview/dashboard/2023-24-pregnant-persons-coverage.html

††
https://www.cdc.gov/rsvvaxview/dashboard/2023-24-nirsevimab-coverage-infants.html

§§
https://www.cdc.gov/vaccines-adults/hcp/imz-standards/

¶¶
https://www.acog.org/programs/immunization-for-women/physician-tools
